# FPGA Integrated Optofluidic Biosensor for Real-Time Single Biomarker Analysis

**DOI:** 10.1109/jphot.2021.3127484

**Published:** 2021-11-15

**Authors:** Mohammad Julker Neyen Sampad, Md Nafiz Amin, Aaron R. Hawkins, Holger Schmidt

**Affiliations:** School of Engineering, University of California Santa Cruz, Santa Cruz, CA 95064 USA; School of Engineering, University of California Santa Cruz, Santa Cruz, CA 95064 USA; ECEn Department, Brigham Young University, Provo, UT 84602 USA; School of Engineering, University of California Santa Cruz, Santa Cruz, CA 95064 USA

**Keywords:** Optofluidics, single molecule detection, biophotonics, field programmable gate array (FPGA), anti-resonant reflecting optical waveguide (ARROW), real-time

## Abstract

Integrated optofluidic biosensors can fill the need for sensitive, amplification-free, multiplex single molecule detection which is relevant for containing the spread of infectious diseases such as COVID-19. Here, we demonstrate a rapid sample-to-answer scheme that uses a field programmable gate array (FPGA) to enable live monitoring of single particle fluorescence analysis on an optofluidic chip. Fluorescent nanobeads flowing through a micro channel are detected with 99% accuracy and particle concentrations in clinically relevant ranges from 3.4×10^4^ to 3.4 × 10^6^/ml are determined within seconds to a few minutes without the need for post-experiment data extraction and analysis. In addition, other extract salient experimental parameters such as dynamic flow rate changes can be monitored in real time. The sensor is validated with real-time fluorescence detection of single bacterial plasmid DNA at attomolar concentrations, showing excellent promise for implementation as a point of care (POC) diagnostic tool.

## I. INTRODUCTION

THE need for sensitive, quick and compact diagnostic platforms for instant screening of infectious diseases has been brought into sharp focus by the COVID-19 pandemic [1], [2]. Integrated microfluidic biosensors can detect different biomarkers, e.g., nucleic acids and proteins, from bodily fluids and have shown potential to be implemented as customized point of care (POC) platforms in case of outbreaks [3]–[7]. For enhanced performance and applications, recently optofluidic devices have been developed exploiting optical properties such as fluorescence [8], [9], Raman scattering [10], and refractive index change [11] combined with different imaging systems [12]–[14]. One of these approaches to an optofluidic analysis platform is based on liquid- and solid-core anti-resonant reflecting optical waveguides (ARROW) and has shown all the advantages of combining microfluidics and optical sample analysis on a single chip [15]–[17]. ARROW devices have shown efficient and amplification-free detection of single bioparticles while they flow through a microchannel under a mechanical or electrical driving force [7], [17], [18]. Moreover, multi-spot excitation for spectrally and spatially multiplexed target detection with excellent signal to noise (SNR) ratio was implemented with multimode interference (MMI) waveguides, [19]–[21]. Moreover, this platform can be easily integrated with a programmable bio sample preparation system for efficient and contamination-free sample transfer process to the sensing region [22], [23]. Short turnaround time is considered as one of the desirable features for a rapid diagnostic tool, but typically signals generated by these aforementioned optofluidic devices need to go through complex signal post-processing steps to produce the desired diagnostic information. In case of a mass testing scheme, these signal post-processing steps also impose delay (at least few minutes) in the time sensitive detection process, usually involving bulk computing resources and, therefore, limited field-deployability. Moreover, existing commercial products [24] for sensitive fluorescence analysis applications such as Fluorescent Lifetime Imaging (FLIM), Förster Resonance Energy Transfer (FRET), or Fluorescence Correlation Spectroscopy (FCS) are expensive and need to be configured for a specific application. A more customized solution that does not rely on the processing speed of the central processing unit (CPU) of a connected computer would be more cost efficient and desirable. Consequently, integration of fast, re-programmable and dedicated electronic processors with these ARROW optofluidic biosensors can overcome these challenges by combining a high-throughput bio-sample detection scheme with a fast and reliable signal processing tool for real-time analysis. Field-programmable gate arrays (FPGAs) which are commonly implemented in embedded systems [25] are ideal for integration with chip-based point-of-care systems due to their reconfigurability, high speed, low power and cost, and compact size. Indeed, FPGA integrated camera based real-time imaging has been implemented for various applications [26], [27], as has been for single molecule detection with avalanche photo detectors(APD)[29].However, a complete real-time bio-sample analysis tool with short turnaround time is yet to be developed. Here, we are introducing an FPGA integrated optofluidic platform for detecting and analyzing individual labeled bioparticles in flow, while extracting the target concentration and other experimental parameters. Real-time implementation enabled by a custom written Verilog program processes signal photon counts hierarchically in three parallel blocks which removes the time constrains of a serial processing scheme. This approach is demonstrated with the real-time analysis of fluorescent nanobeads and individual bacterial plasmid DNAs. A comparison with MATLAB based post-processing analysis shows reliable target detection with 99% accuracy. Moreover, the real-time detection of each target enables accurate determination of clinically relevant concentrations between 3.4×10^4^ and 3.4×10^6^/mL within seconds to a couple of minutes. A model for the settling time to reach a reliable concentration value agrees well with the experiment. Finally, we demonstrate the determination of dynamic changes in the particle flow rate as a result of mechanical pressure changes, illustrating the ability to monitor and adapt experimental parameters on the fly.

## II. EXPERIMENTAL PLATFORM DESIGN

**Fig. 1.**
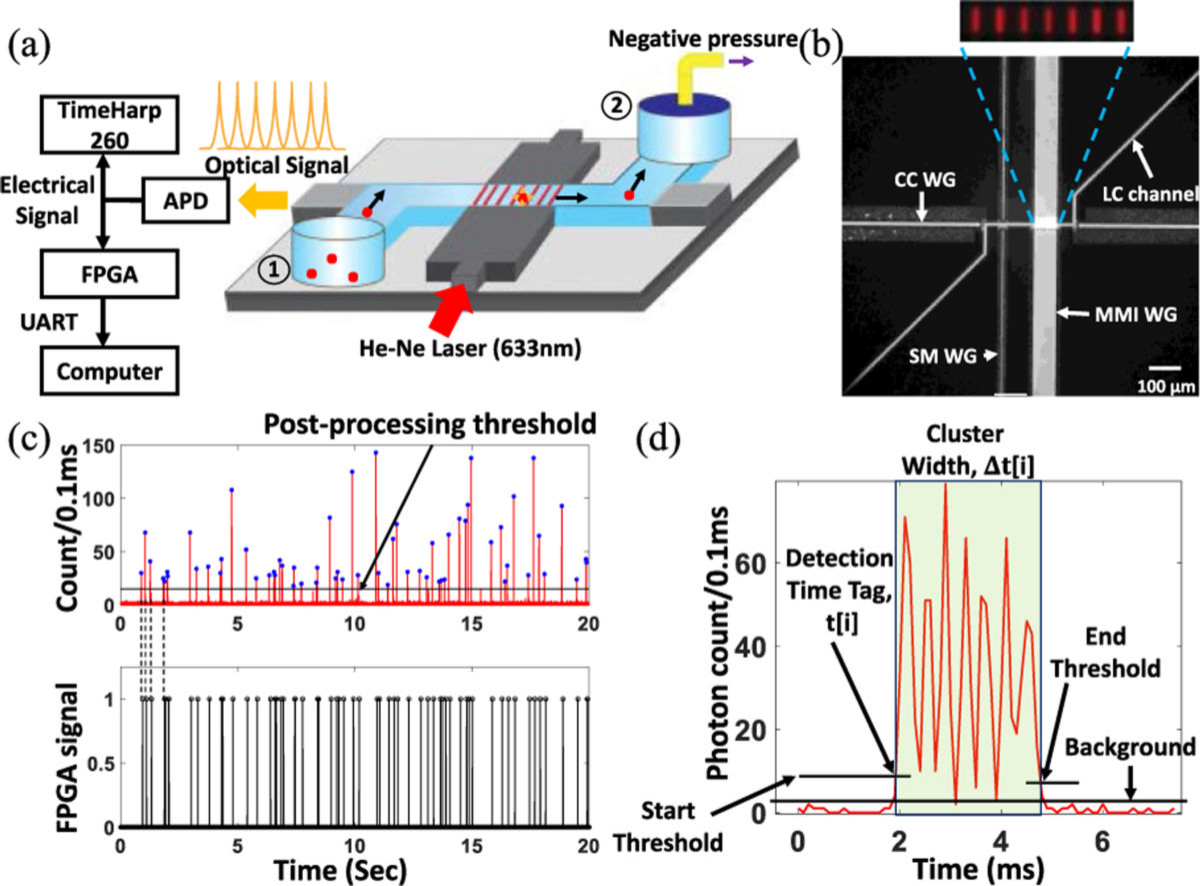
FPGA integrated real-time particle flow analysis platform based on ARROW optofluidic devices. (a) Schematic view of the FPGA integrated ARROW optofluidic device for fluorescence signal collection and analysis. (b) Optical microscope image of the ARROW optofluidic device with connected single-mode (SM), multimode interference (MMI), collection core (CC) waveguides (WG) and liquid-core (LC) channel (Top-down view). Scale bar: 100 *μ*m. Inset shows a 7 spots pattern in the liquid core channel (false colored) when the MMI waveguide is excited with He-Ne laser (633 nm). (c) 200 nm diameter fluorescent bead detection in real-time. Top: detected fluorescence signal with post-processing threshold for conventional signal analysis. Bottom: FPGA confirmation signal for each particle detection event shows excellent one to one correspondence (Dashed lines illustrate the agreement). (d) Zoomed-in view of the detected fluorescence signal from a single 200 nm bead. The peak analysis algorithm is triggered at the detection time tag, *t[i]* and the duration of the detection signal is stored as cluster width, Δ*t[i]*. The background photon count, signal start and end threshold levels (solid black lines) are set as user defined parameters.

Fig. 1(a) shows the experimental platform, which is designed for analyzing an optical fluorescence signal from individual fluorescent target particles in real-time. At the heart of this platform is an Si-based optofluidic chip which allows light guiding through the low refractive index liquid core (LC) channel based on the anti-resonant reflecting optical waveguide (ARROW) principle [16]. The 5 μm × 12 μm liquid channel is terminated by two fluidic reservoirs. These reservoirs allow particle introduction inside the channel and the application of a mechanical driving force to move them in a certain direction. A wide solid core (SC) multimode interference (MMI) waveguide orthogonally intersects the liquid channel at a specific distance (*L*) and creates a multi-spot excitation pattern where the number of spots (*N*) is determined by

(1) $$N\lambda =\frac{nW^{2}}{L}$$

Where, λ is the excitation wavelength, *W* the width and *n* the refractive index of the SC MMI waveguide material.

The MMI waveguide in the ARROW optofluidic platform displayed in Fig. 1(a) is designed accordingly (*L* = 1975 μm, *W* = 75 μm, *n* = 1.51) to create a *N* = 7 spots excitation pattern when it is excited by a λ = 633 nm wavelength He-Ne laser beam. Fig. 1(b) shows the top-down view of the intersecting LC channel and SC waveguides. The inset shows the zoomed-in view of the 7 spots excitation pattern (false colored) projected on the LC channel when the channel is filled with quantum dot solution and excited with 633 nm He-Ne laser light. When a negative pressure is applied through the metal reservoir attached to the LC channel outlet, it drags the optically labeled target particle suspended inside the inlet fluid reservoir through the LC channel towards the outlet. The particle passes through the excitation spots (here, seven spots) and emits fluorescence signal which is guided through the LC channel governed by the ARROW principle and collected by a solid core (SC) collection waveguide. Therefore, each particle detection event produces a cluster of uniformly spaced 7 peaks signal in time domain. The collected optical signal is then filtered and converted to an electronic pulse signal by an avalanche photodetector (APD). The APD has single photon counting sensitivity and creates a transistor-transistor logic (TTL) pulse when it detects a photon. For the real-time analysis scheme, the APD output pulse signal is connected to a field programmable gate array (FPGA) (Xilinx Artix-7 FPGA AC701 Evaluation Kit), which is programmed with a custom written verilog code for acquiring and analyzing the input electronic signal for detecting the salient features of the collected fluorescence signal (The detail algorithm is explained in the “Algorithm Design” section). The results of this detection schemearetransferredthroughuniversalasynchronousreceivertransmitter (UART) communication protocol and displayed on a terminal emulator (Tera Term Version 4.101, open source) while the experiment is still running. Another branch of the APD output electronic pulse signal is connected with a time-correlated single photon counting (TCSPC) and multi-channel scaling (MCS) device (TimeHarp 260, Picoquant) which stores the photon arrival time. The stored data can be utilized for signal post-processing and comparing the performance of the FPGA based real-time particle detection and flow analysis scheme.

## III. ALGORITHM DESIGN

In order to detect the fluorescence signal from the labeled particle in real time, an FPGA is connected with the APD and a custom Verilog code is used for processing and calculating essential parameters from the collected and filtered raw optical signal (See supplementary information S1: Fig. S2 and S3). The complete workflow of the code is divided into three independent blocks; acquisition, processing, and display block with pre-defined hierarchy (See supplementary information S1: Fig. S1). The acquisition block is the upper-level block which is designed to acquire and count the TTL pulse signal from the APD, which corresponds to every single photon detected by the photo sensitive semiconductor photodiode. A sequential counter inside the acquisition block counts the number of photons within a user defined binning time. As each TTL pulse corresponds to a single photon, the binned count corresponds to the intensity of the collected fluorescence signal by the collection core waveguide. The binning time is chosen carefully so the signal is not under or over sampled. In the case of undersampling, the shape of the binned photon count histogram will not be able to reveal the accurate shape of the fluorescence signal with 7 individual peaks. Whereas, for the oversampling case the signal level will be too low and the difference between consecutive data points will not be considerable. For all the experiments described here, the binning time is 100 μs so the integrated photon counts can properly reconstruct the 7 peaks fluorescence signal. After the binning time, this upper-level block triggers the mid-level processing block and transfers the binned photon count. The acquisition block again starts to acquire and count the number of photons for the next binning interval without waiting for other lower-level blocks to complete their functions. The processing block is the brain of the whole system and is designed by following a finite state machine (FSM) model (See supplementary information S1: Fig. S4). When a target particle enters the MMI wave guide excitation region, it generates a fluorescence signal consisting of multiple peaks based on the excitation spot pattern. The main function of this block is to detect the cluster and calculate the position and width of each peak in the time domain with respect to the beginning of the experiment. Though the workflow of this block starts after being enabled by the upper-level data acquisition block, it can run for multiple cycles by itself before it finishes the required calculation. After receiving the binned photon count, the block compares the difference between consecutive data points with a pre-defined threshold value. If the threshold is crossed, it triggers either a cluster or peak start detection state inside the FSM. Then the height and the width of each peak are calculated. There is a user defined wait time (usually 1 ms) after the signal goes below a threshold value, which indicates the ending of the detected peak. The wait time helps to determine the end of the detection signal cluster and is chosen in a way that it is longer than the usual time difference between two consecutive peaks in a cluster but shorter than the mean time difference between two particle detection events. It is worth noting that the platform is designed for single molecule analysis at clinically relevant target concentrations(C≤10^6^/mL). If multiple particles enter the excitation region simultaneously or within a time limit (related to the volumetric flow rate during the experiment), which may happen at a higher target concentration (approximately C *>* 10^8^/mL), the designed platform will consider them as a single particle detection event. However, this limitation can be overcome by decreasing the excitation volume or pre-diluting the target solution. After confirming a successful target particle detection event, the processing block calculates the flow-based parameters such as target particle concentration and triggers the display block which has the lowest priority. While triggering the display block, the processing block transfers the calculated results before the corresponding registers are updated for the next computational cycle. We have implemented UART communication between the FPGA and the data receiving terminal installed on a personal computer. To have a fast data transfer process we have utilized a custom base 64 encoding scheme and transferred encoded data at 921600 /s baud rate. The display block can transfer user defined number of variables to the terminal. We have chosen four important variables i.e., the number of detected particles, time tag of the detection event, MMI optical signal cluster time duration and calculated cumulative mean concentration of the target particles to be displayed. It takes ∼271.26 μs to display 25 characters line in the terminal emulator which is well below the mean duration between two consecutive particle detection events combined with the cluster end confirmation wait time. It is also possible to connect a custom LabVIEW program with the FPGA for faster data transfer and to store the displayed results in a computer data storage. Furthermore, the stored results can be easily uploaded to the cloud or shared with a server for remote accessibility.

## IV. RESULTS AND DISCUSSION

In order to characterize the performance of the FPGA based real-time particle detection scheme, initially 200 nm diameter fluorescent beads (FluoSpheresTM, 625/645nm Crimson) at 3.4×10^6^/mL concentration were driven through the LC channel and excited by the 7-spot MMI pattern. A He-Ne laser at 633 nm wavelength (P = 4 mW) is used as light source. Each MMI spot has an approximate excitation volume of 69 fL with 15.4 μW average excitation power. The collected fluorescence signal was analyzed with FPGA based algorithm in real-time and the result was displayed upon the confirmation of the particle detection process. A comparison between post-processed signal and the corresponding detection confirmation signal obtained in real-time shows excellent one to one correspondence (Fig. 1(c)). The accuracy of the FPGA based real-time analysis method was calculated to be 99% with only 5 missing peaks among 472 particle detection events. When the signal strength is weak, the peak height becomes comparable to the threshold value and the peak can be missed by the FPGA circuit. This issue can be addressed by calculating the threshold automatically based on the background photon count at the beginning of the experiment.

The collected fluorescence signal was further analyzed for determining different temporal features of the detected signal cluster. Fig. 1(d) shows a zoomed-in view of a 7 peaks cluster where background photon count and the user defined threshold values are displayed. The particle arrival time with respect to the beginning of the experiment (Detection Time Tag (*t[i]*)) is determined by comparing the binned photon count with the cluster start threshold and the cluster end threshold was utilized for determining the fluorescence signal width of the same particle in time domain (Cluster Width (Δ*t[i]*)). Furthermore, the time tags of each single peak comprising the cluster were calculated based the Verilog algorithm. All these time domain data were utilized to reveal important flow parameters of each detected particle such as individual particle velocity, volumetric flow rate, and detection rate.

**Fig. 2.**
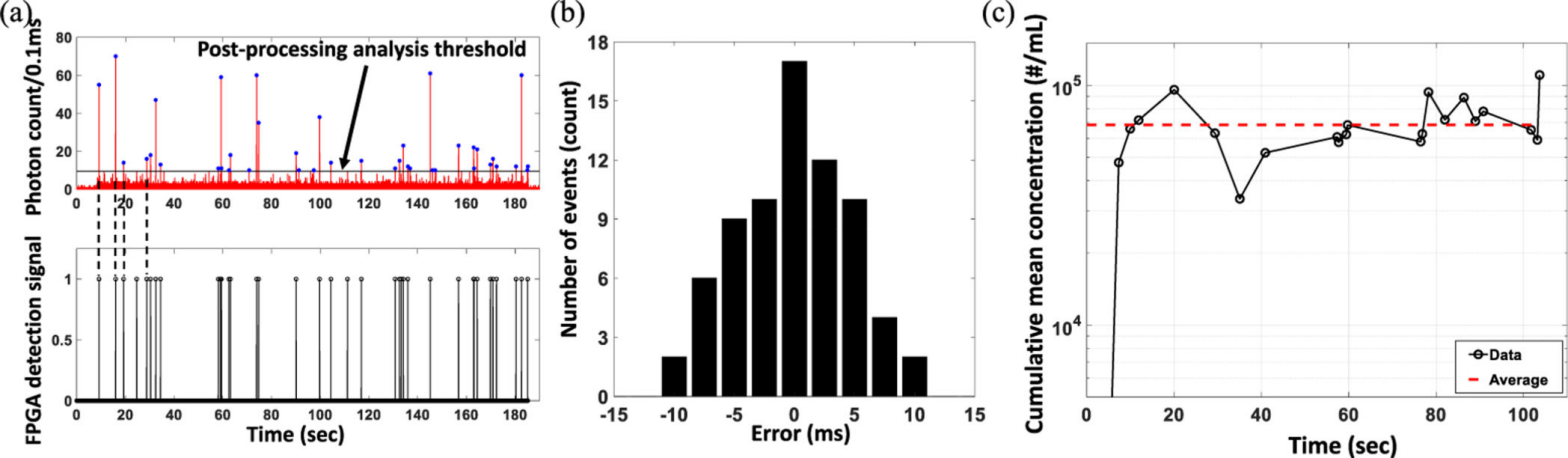
Real-time flow analysis of dye labeled NDM plasmid. (a) One to one correspondence between post-processed fluorescence signal (top) and real-time FPGA event detection signal (bottom). (Dashed lines illustrate the agreement). (b) Relative error histogram of the particle detection time calculated by the live FPGA analysis algorithm in comparison with the MATLAB post analysis. (Sample size, n = 72). (c) Calculated cumulative mean concentration of the target sample with experiment time. Horizontal dashed line (red) shows the average value of the calculated cumulative mean concentration values (*Cave*), which is 6.848 X 10^4^/mL or 113 aM.

To demonstrate the sensitivity of the real time detection algorithm, the system was tested with fluorescently labeled biological sample. Specifically, a double stranded (ds) DNA sample corresponding to an antibiotic resistant pUC19- New Delhi metallo-*β*-lactamase (NDM) plasmid was prepared by sequence specifically extracting the target DNAs from the blood sample spiked with E. coli cells having pUC19-NDM plasmids and then labeled with nucleic acid staining dye. The target sample was prepared with a multi component system, consisting of cell extraction from the whole blood, nucleic acid sample preparation in a microfluidic chip [21]. While the initial plasmid concentration spiked into the starting amount of 7mL whole blood was known (5 ×10^6^ CFU mL^−1^), there was non-trivial loss of DNA targets before the sample was introduced in the ARROW chip, in particular in the intermediate sample prep chip. Therefore, the actual concentration that was optically detected was not known. Finally, the plasmid sample was stained with Syto 62 red fluorescent intercalating dye (Ex/Em: 649/680nm). The nucleic acid staining dye has high affinity for nucleic acids and becomes fluorescent upon binding to the plasmids. The target staining process was validated by blank control experiments (See supplementary information S2: Fig. S5). This prepared solution at unknown concentration were driven through the LC channel and analyzed in real-time. Fig. 2(a) illustrates good one to one correspondence between both detection mechanisms for single plasmid DNA detection.

To further quantify the accuracy of the real time detection scheme, the corresponding particle detection time tags (illustrated in Fig 1(c)) were compared. The error histogram shows ∼80.6% of the total detected events reside within a relative error band of ±5 ms. This error can be generated from the noise induced in the APD electric pulse signal while transferring through the BNC connector as well as the voltage drop due to multiple branch circuitry. Also, there is a finite time mismatch between the starting time of the binning step of both analysis mechanism, which yields different binned photon count at the same time stamp. Due to a combination of these factors, binned optical signals in real time analysis and post processing pass the starting threshold at two different times, eventually generating a time discrepancy between the detection time tags. Regardless, the system shows great robustness with majority of the events detected within very small deviation.

Next, the concentration of the fluorescent targets was determined which is one of the unique and essential features of this real-time particle flow monitoring platform. The cumulative mean concentration (*C*) is calculated by utilizing the detected flow parameters such as, particle velocity, volumetric flow rate and instantaneous concentration (c_i_) shown in (4).

(2) $$v(i)=\frac{W}{\text{Δ}t(i)}$$

(3) $$c_{i}(i)=\frac{i}{v(i)\cdot A\cdot t(i)}$$

(4) $$C(i)=\frac{\left[\sum_{j=1}^{j=i}C_{i}(j)\right]}{i}$$

Here, *i* is the detected event number from the beginning of the experiment, *v(i)* is the instantaneous particle velocity, *W* is the width of the MMI waveguide, *A* is the cross-sectional area of the LC channel, *t(i)* and Δ*t(i)* are the detection time tag and the cluster width of the detected fluorescence signal, respectively (see Fig. 1(d)).

Fig. 2(c) shows the calculated cumulative mean concentration of the NDM plasmid solution from the extracted time domain information by FPGA from the optical trace showed in fig. 2(a). The detected average concentration (*C*_*ave*_) of the bacterial plasmid DNA sample was 113 aM (see red dashed line in fig. 2(c)). This demonstrates the applicability of our designed real-time platform for detecting single biomolecules and their concentration, and is the principal result of this letter.

**Fig. 3.**
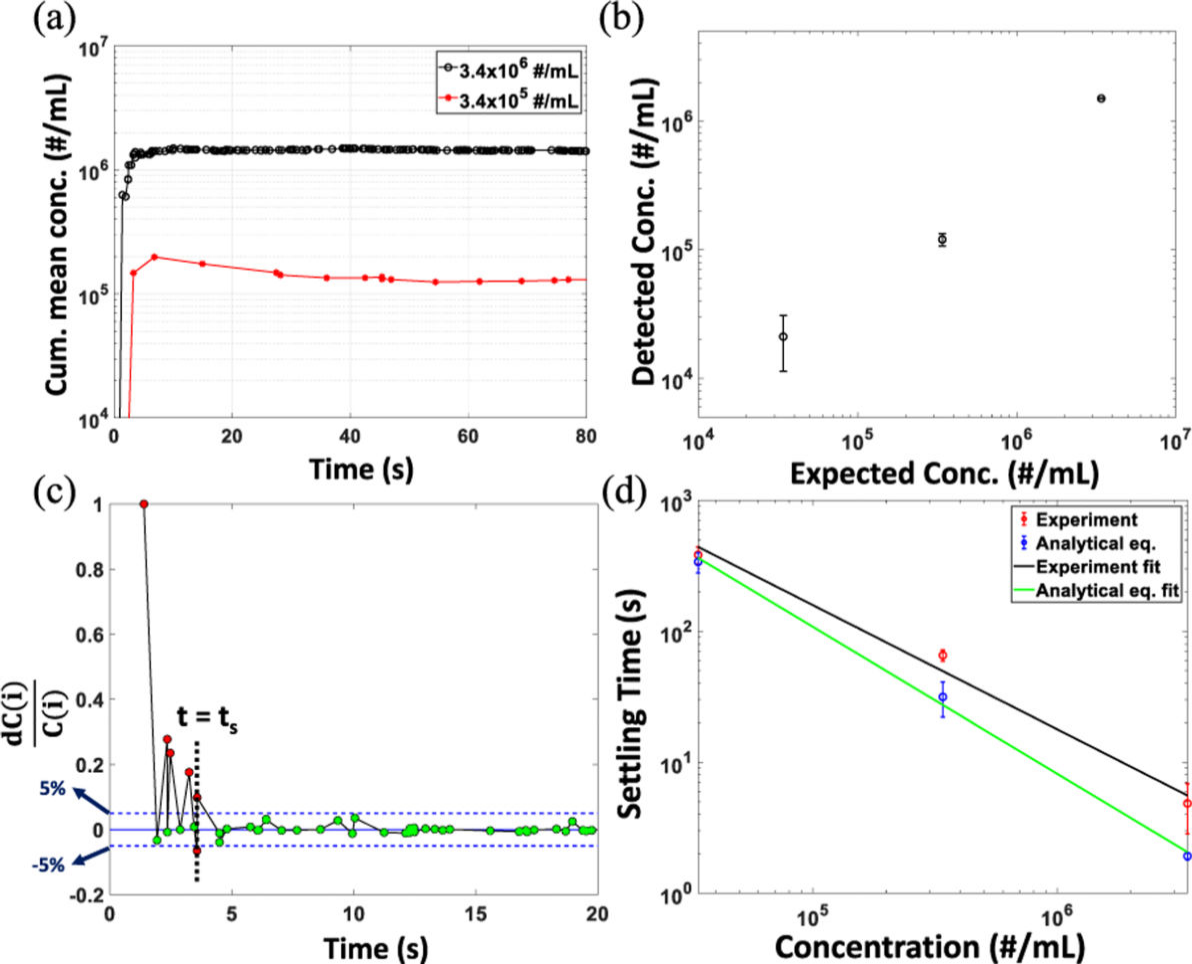
Real-time particle concentration detection by FPGA integrated analysis platform. (a) Cumulative mean concentration of 200 nm fluorescent bead solution detected in real-time. (b) Real-time detected vs expected particle concentration. (Error bars: standard error of mean.) (c) Relative error of detected cumulative mean concentration vs time. The horizontal dashed lines (blue) show the ±5% deviation window and the vertical dashed line (black) show the settling time, *ts*. (d) Settling time calculated in real-time and in post-processing with constant volumetric flow rate consideration at different target concentration with linear fit, R^2^ = 0.98 and R^2^ = 0.99 respectively.

In order to further illustrate the reliability and repeatability of the detection scheme, we present comparison studies between expected and real-time calculated target concentration values using the same 200 nm fluorescent beads detected in Fig. 1(c) with concentrations between 3.4×10^4^/mL and 3.4×10^6^/mL. Fig. 3(a) shows how quickly the cumulative mean concentration values changed and stabilized as the experiment progressed. The detected concentration is slightly lower than the input concentration because of imperfect collection of the low fluorescence signal generated from the beads near the channel walls. Statistical analysis was performed on these real-time concentration data to find out the time required for this platform to provide stable and reliable analysis results. This information can also be utilized to optimize the experiment run time for unknown concentration sample analysis. The repeatability of the real-time calculation process is displayed in Fig. 3(b), showing good agreement between calculated and measured values over the entire concentration range. As for the higher concentration solution (3.4×10^6^/mL), the particle detection rate was higher and the deviation from the calculated concentration values was smaller compared to the values obtained at 3.4×10^4^/mL.

Furthermore, statistical analysis was performed on the time versus cumulative mean concentration data traces to extract the settling time for the analysis platform at a particular target concentration. The settling time is defined as the time form the beginning of the experiment after which the concentration remains stable within a ±5% window. Fig. 3(c) shows the settling time for the platform at target concentration 3.4×10^6^/mL. The relative concentration deviation is calculated by (5).

(5) $$\frac{dC(i)}{C(i)}=\frac{C(i)-C(i-1)}{C(i)}$$

Where, *C(0)* = 0 and *i* is the detected event number from the beginning of the experiment. Considering constant volumetric flow rate, (5) can be simplified as shown in (6).

(6) $$\frac{dC(M)}{C(M)}=1-\frac{M}{M-1}\left[\frac{\sum_{1}^{M-1}\frac{\text{i}}{\text{t}(\text{i})}}{\sum_{1}^{M}\frac{\text{i}}{\text{t}(\text{i})}}\right]$$

Here, *M* is the number of detected particle events corresponding to the settling time (*t*_*s*_). Although no analytical solution for *M* exists, the most striking behavior of the value for *M* is that it remains within an upper limit, here 15, irrespective of the target concentration. As a smart and efficient microfluidic platform, this settling time information can be easily utilized to design the experiment running time for getting statistically reliable result.

Fig. 3(d) shows how the settling times calculated in real-time and in post-processing with constant volumetric flow rate (according to (6)) are related to the target concentration. As any fluctuation of the cumulative mean value depends on previous data points, at a higher concentration the count rate is higher, leading to quicker stabilization and shorter *t*_*s*_. It is evident from the inverse relation between the settling time and the target concentration displayed in Fig. 3(d). We note that the settling time calculated with simplified analytical equation is shorter compared to the ones calculated in real-time, as the volumetric flow velocity of the liquid along the rectangular cross section channel is not entirely constant.

**Fig. 4.**
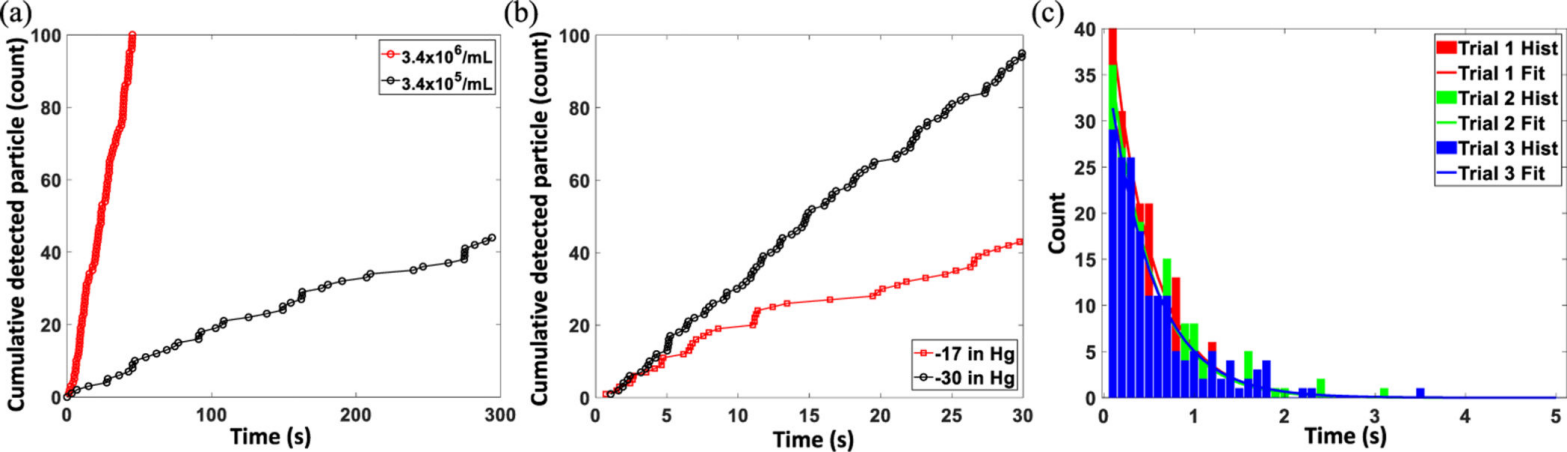
Particle detection rate and event frequency analysis. (a) Variation of the particle detection rate with target concentration. (b) variation of the particle detection rate with the magnitude of applied negative pressure. (c) Event frequency analysis. Histogram of the time between successive events at target particle concertation 3.4×10^6^/mL (solid line: exponential fit).

We also investigated how experimental parameters can affect the particle detection rate and real-time monitoring of the particle detection events can be utilized. Fig. 4(a) shows the variation in particle detection rate when the target particle concentration was changed from 3.4×10^5^/mL to 3.4×10^6^/mL. It is evident that at a lower concentration, the approximate slope of the plot is less steep as the number of detected events within a fixed time becomes smaller. Also, the time difference between two consecutive events increase and become more erratic. Similar effects were observed when the magnitude of the applied negative pressure was changed. Fig. 4(b) is showing that the same target solution can have lower detection rate when a weaker driving force is applied e.g., due to valve actuation or unintentional pressure fluctuations. Another issue regarding the particle flow is that the LC channel inlet can be clogged by unwanted residue or precipitations from the target sample. In this case, particle flow will also be unintentionally interrupted, and the flow-based analysis results will be erroneous. This real time particle detection event monitoring can easily help researchers to identify these issues quickly and to take immediate action so valuable reagents can be saved. Finally, a statistical analysis was performed based on the time difference between two consecutive detected events. Fig. 4(c) displays the histogram analysis of the time between successive events with 0.1 s binning time and it shows an exponential decay as expected for a Poisson process with independent bead arrival time. The probability distribution function for the arrival of a bead in time *t* after the previous one is *P(t) α e*^−λ*t*^, where λ is mean arrival rate. The analysis was repeated three times, and indicates excellent reproducibility between assays.

## V. CONCLUSION

In summary, we have introduced FPGA control of an integrated ARROW optofluidic platform for live signal processing and particle flow analysis. First, a detection accuracy of 99% in comparison with post-processing analysis has been demonstrated in real-time with both fluorescent nanobeads and nucleic acid biomarkers. The approach enabled determination of the target concentration on the fly within seconds to minutes. Additionally, statistical analysis showed reliability and reproducibility of the detection process. Finally, live monitoring of the target detection rate on this platform can be utilized for efficient delivery of target particles to the sensing region without wasting valuable reagents. In the future, this platform can be configured with different ways to generate excitation spot patterns such as y-splitters [20] or top-down excitation [28] as well as machine learning algorithms for better detection efficiency and accuracy [29]. Integration of an electrical feedback circuit for smart particle delivery system (variable particle driving force) based on the real-time flow analysis result is also feasible.

## Supplementary Material

supp1-3127484
