# Supplementary material for: FPGA Integrated Optofluidic Biosensor for Real-Time Single Biomarker Analysis
Source: IEEE Photonics J. Author manuscript; Available in PMC 2022 Feb 1. (PMC8658630; doi:10.1109/jphot.2021.3127484)
Supplement: supp1-3127484 [file NIHMS1760396-supplement-supp1-3127484.docx]

**Supplementary information for**

**FPGA Integrated Optofluidic Biosensor for Real-time Single Biomarker Analysis**

Mohammad Julker Neyen Sampad^1^, Md Nafiz Amin^1^, *Student Member, IEEE*, Aaron R. Hawkins^2^, *Fellow, IEEE*, and Holger Schmidt^1^, *Fellow, IEEE*

^1^School of Engineering, University of California, Santa Cruz, 1156 High Street, Santa Cruz, CA 95064, USA

^2^ECEn Department, Brigham Young University, Provo, UT 84602 USA

**Supplementary Information:**

**Supplementary Fig. S2.** Screenshot of Xilinx Vivado 2015.2 software showing I/O planning of the programmed FPGA

**S1. Details of the FPGA circuit and the custom written Verilog program**

**Supplementary Fig. S1.** Simplified block diagram of the Verilog program.

**Supplementary Fig. S3.** Photograph of FPGA circuit and electronic connection.

**Supplementary Fig. S4.** Simplified finite state machine (FSM) diagram for the processor block of the Verilog program.

**Supplementary Fig. S5.** The blank trace was done by mixing nucleic acid staining dye (1uM of Syto62) with a blank sample (only 1XPBS) without any plasmid target present. ). In the absence of plasmid DNA, the dye didn’t generate fluorescence signal. The fluorescence trace detected using ARROW optofluidic chip show no signal was above the set threshold 10 counts/0.1ms (blue solid line).

no signal above the set background threshold of 11 counts/0.01ms.

**S2. Fluorescence signal from blank trace**
